# Dyslipidemia in Lebanese children and adolescents: parents’ knowledge and practices

**DOI:** 10.1186/s12889-026-28254-8

**Published:** 2026-06-24

**Authors:** Khalil Hamadeh, Mariana Boulos, Bachir Atallah, Maya Bou Hamdan, Dina Maddah, Nada Ghorayeb, Marie-Hélène Gannagé Yared

**Affiliations:** 1 Department of Endocrinology at Hôtel-Dieu de France Hospital, Faculty of Medicine, Saint-Joseph University, Beirut, Lebanon; 2 Clinical Research Center, Faculty of Medicine, Saint-Joseph University, Beirut, Lebanon

**Keywords:** Dyslipidemia, Children, Adolescents, Lebanese, Parents, KPA

## Abstract

**Background:**

Pediatric dyslipidemia is a common metabolic disorder worldwide, especially in Lebanon, and is an early risk factor for cardiovascular disease. Parents play a crucial role in prevention, screening, and management, but their knowledge and practices remain poorly studied.

**Methods:**

A cross-sectional questionnaire-based study was conducted between March and November 2025 among parents of school-aged children from eight schools in Greater Beirut and Mount Lebanon. An electronic questionnaire was used to collect sociodemographic data and to assess parents’ knowledge (using a composite score) and practices regarding dyslipidemia. Factors associated with knowledge levels and their relationship with parental practices were also examined.

**Results:**

A total of 946 parents were included (861 women and 85 men) with a mean age of 43.9 ± 6.2 years, 94.1% had a university education, and 11.6% were healthcare professionals. Among them, 42.4% had heard of pediatric dyslipidemia, mainly through the media (25.2%) and healthcare professionals (24.4%). The mean knowledge score was 14.1 ± 4.4 out of 23. Blood test-based lipid screening for dyslipidemia in children had been performed by only 6.7% of parents. The independent determinants of a higher knowledge score were female gender, university education, healthcare professional status, and having heard of pediatric dyslipidemia (*p* < 0.001, *p* = 0.007, *p* < 0.001, *p* < 0.001, respectively). In addition, higher knowledge scores were significantly associated with favorable preventive practices, including implementation of healthy eating habits (*p* = 0.02) and lipid screening in children (*p* < 0.001).

**Conclusion:**

Parents demonstrated moderate knowledge levels. The association between knowledge level and the adoption of favorable preventive measures suggests that educational campaigns targeting parents and health care professionals could play a role in promoting healthier practices.

**Supplementary Information:**

The online version contains supplementary material available at https://doi.org/10.1186/s12889-026-28254-8.

## Highlights

Awareness and knowledge of dyslipidemia in children among Lebanese parents are unknown.

Only a small proportion of parents reported lipid screening in their children.

Female gender, higher education, and healthcare professional status were independent predictors of better knowledge.

Higher knowledge was associated with favorable preventive practices, including healthier dietary habits and lipid screening.

These findings highlight the potential value of targeted educational interventions, which may help support early cardiovascular risk prevention in children.

**Supplementary Information:**

The online version contains supplementary material available at https://doi.org/10.1186/s12889-026-28254-8.

## Introduction

Dyslipidemia is a common metabolic disorder characterized by high levels of low-density lipoprotein cholesterol (LDL-C), high triglyceride (TG) levels, low high-density lipoprotein cholesterol (HDL-C) levels, and/or high non-HDL-C cholesterol levels [1]. It can be primary, associated with genetic abnormalities, mainly represented by familial hypercholesterolemia [2], or secondary to medical conditions ( e.g., obesity, diabetes, and hypothyroidism) or medication use [3].

In the United States (US), approximately one in five children and adolescents has dyslipidemia with abnormal total cholesterol (TC), HDL-C, or non-HDL-C levels [4]. This prevalence increases with age, with 15.2% of children aged 6 to 11 years and 25.2% of adolescents aged 12 to 19 years having at least one adverse lipid level [5]. In the first Lebanese study, the prevalences of high non-HDL-C levels (> 3.8 mmol/L), very high non-HDL-C levels (> 4.9 mmol/L), and high TG levels among schoolchildren were respectively 9.2%, 1.24%, and 26.6% [6]. In another study performed on Lebanese children aged between 2 and 10 years, the prevalences of high TC, non-HDL-C, LDL-C, TG, and low HDL-C levels were 19.5%, 23%, 19%, 31.8%, and 20%, respectively [7].

The *National Heart*,* Lung*,* and Blood Institute* (NHLBI), supported by the *American Academy of Pediatrics* (AAP), recommends universal lipid screening between the ages of 9 and 11 years, then again between 17 and 21 years [8]. It also advocates targeted screening starting from the age of 2 years in children with risk factors, such as a family history of premature cardiovascular disease (CVD) or dyslipidemia, obesity, hypertension, or diabetes [8]. This screening aims to identify children at increased risk of developing CVD in adulthood [9], as abnormal lipid levels during childhood are associated with a higher risk of developing atherosclerosis and CVD in early adulthood and midlife [10, 11]. On the other hand, the *U.S. Preventive Services Task Force (USPSTF)* and *the American College of Family Physicians* believe that there is insufficient evidence to assess the benefit-to-risk ratio of screening for familial dyslipidemia in children and adolescents under 20 years of age [12]. Therefore, even if current guidelines provide overall consistent recommendations for the management of childhood dyslipidemias, there is a lack of consensus regarding universal screening for early detection of lipid disorders [13].

Numerous studies have assessed adults’ knowledge and awareness of dyslipidemia [14–18]. These studies were conducted in the US [14], Europe [15], Korea [16], China [17], and Saudi Arabia [18]. Across all settings, patient awareness of dyslipidemia was generally low, ranging from approximately 15% in China [17] and Luxembourg [15] to 21% in the US (the Bogalusa Heart Study) [14], and reaching 58.5% in a Korean study [16]. Older age [14, 15], a higher educational level [14], positive family history of coronary artery disease [14], and higher body mass index [15] were significantly associated with greater awareness of dyslipidemia.

However, parents’ knowledge and practices regarding dyslipidemia in children have been poorly studied, with a limited group of subjects included in the studies. In a US study performed on 91 parents, 45.5% were in favor of universal screening, and 48.4% preferred targeted screening based on the child’s personal and family history [19]. In a second Irish study, 98% of 53 parents of hospitalized children agreed to have their children screened [20]. To our knowledge, no other study has assessed parents’ knowledge and attitudes regarding pediatric dyslipidemia. Because children depend largely on their parents for access to preventive health care, parental awareness and attitudes may play an important role in the acceptance of lipid screening, follow-up of abnormal results, and adoption of lifestyle changes. A broader study assessing parents’ knowledge and practices regarding this subject would help identify existing gaps and inform the development of targeted interventions to encourage early screening.

The present study aims to assess the knowledge and practices of Lebanese parents regarding pediatric dyslipidemia and to identify the factors that might affect knowledge.

## Materials and methods

This is a cross-sectional survey-based study conducted between March and November 2025 among parents of children enrolled in eight Lebanese private schools from the Greater Beirut and Mount Lebanon regions. The participating schools included primary, intermediate, and secondary levels; therefore, the children represented in the study ranged in age from 5 to 18 years.

### Sampling strategy

The study used a school-based, non-random sampling strategy. Eight private schools located in Greater Beirut and Mount Lebanon (GB/ML) were selected from a list of eligible schools using a purposive, convenience-based approach. GB/ML area was selected because it concentrates the majority of students, particularly after the war period. School selection was based on administrative approval, ease of access, number of enrolled students, and socioeconomic profile. To improve socioeconomic variability within the private-school population, the three schools with the highest number of students from higher socioeconomic backgrounds and the five schools with the highest number of students from low-to-middle socioeconomic backgrounds were invited and included. Public schools in the targeted areas were not included because they comprise a highly heterogeneous student population, including a substantial proportion of non-Lebanese refugee students. Since the present study aimed to assess parental knowledge and practices among Lebanese parents and given that public schools account for less than 25% of enrolled students in Lebanon, recruitment was limited to private schools to maintain a more homogeneous study population.

### Preparatory phase

Before distributing questionnaires, meetings were held with the administrations of each participating school to present the study’s objectives and provide a brief overview of dyslipidemia. Following review by the school medical teams, including the pediatrician and the school nurse, the questionnaire was approved by the school’s administration. The study and questionnaire were also presented to each school’s parents’ committee to encourage parental participation and response.

### The questionnaire

The participant information sheet and questionnaire available in English, French, and Arabic are provided as supplementary material. Both documents were developed specifically for the present study and have not been previously published. The questionnaire uses simple language to ensure comprehension across different educational levels. The participant information sheet explained to parents the purpose of the study, the voluntary nature of participation, and the confidentiality of responses. It also provided a brief explanation of dyslipidemia, defined as abnormal blood lipid levels, including elevated cholesterol, triglycerides, or abnormal high-density lipoprotein (HDL) levels. Participants were informed that completion of the questionnaire implied consent. The questionnaire was developed specifically for this study after reviewing the available literature on pediatric dyslipidemia, cardiovascular risk factors, screening recommendations, prevention, and parental health-related knowledge and practices. It was reviewed by three pediatricians, who evaluated the relevance, clarity, and comprehensiveness of the items and provided qualitative feedback. Based on their comments, several items were reworded to improve clarity and clinical accuracy. The questionnaire was then pilot-tested with 30 parents to assess its comprehensibility. These 30 parents were excluded from the final analysis and were not included in the final sample of 946 participants. Participants were asked to comment on the wording of the items, ease of understanding, and overall satisfaction. Based on their feedback, minor modifications were made to improve clarity. The questionnaire was then distributed via Google Forms to parents through the school administrations, mainly via official school communication channels, including school WhatsApp groups and/or electronic messages (via the school Microsoft Teams). All parents of students enrolled in the participating schools were eligible to participate. Participation implies that parents were able to understand and complete the online questionnaire and provided informed consent. No specific exclusion criteria were applied, particularly regarding the child’s medical history, parental medical background, or previous exposure to health education diseases on dyslipidemia. The response rate was approximately 25%, which is acceptable for a voluntary, school-based online questionnaire addressed to parents.

The questionnaire was available in three languages (French, English, Arabic) to optimize response rates and underwent a translation and back translation process [21]. The internal reliability of the questionnaire was assessed using Cronbach’s alpha to evaluate the consistency between the knowledge items. The results showed good internal consistency, with a Cronbach’s alpha coefficient of 0.76., Kaiser-Meyer-Olkin measure (= 0.717) and Bartlett’s test of sphericity (*p* < 0.001).

The questionnaire consists of four main parts:

1. Sociodemographic and personal characteristics of parents:
  This section assessed the following variables: age, gender, level of education (primary, secondary, or university), professional status (healthcare professional or non-healthcare professional), and number of children.
2. Parental awareness about dyslipidemia
  This section consisted of three questions: the first assessed prior awareness of dyslipidemia in children; the second examined participants’ sources of information; and the third evaluated their self-reported level of knowledge on the topic.
3. Knowledge about pediatric dyslipidemia:
  This section includes seven questions on dyslipidemia knowledge. Knowledge was assessed using a total score out of 23 points. The score was calculated by awarding 1 point for each correct answer and 0 points for incorrect answers. The score is assigned according to the following method:
    ◦Three questions concerning the need for dyslipidemia screening, the optimal age to begin screening, and the method of diagnosing dyslipidemia (3 points in total).
    ◦ Four questions on risk factors, diseases that cause dyslipidemia, management practices, and preventive strategies. Each question has five correct answers, each worth 1 point, for a total of 20 points.
  The knowledge score was analyzed as a continuous quantitative variable.
4. Screening practices and prevention habits:
  This section evaluates parental practices for screening their children for dyslipidemia, as well as preventive habits such as encouraging physical activity and adopting a balanced diet rich in fruits and vegetables.

### Statistical analysis

Qualitative variables were described as frequencies and percentages. Quantitative variables were described using means and standard deviations (M ± SD) (since their distribution was normal).

Associations between the knowledge score and sociodemographic variables (gender, age, education level, healthcare professional status, number of children, prior exposure to information on dyslipidemia) were explored using parametric tests (Student’s t-test, ANOVA test). The correlation between age and knowledge score was assessed using Pearson’s correlation test.

Associations between the knowledge score and parental preventive practices (use of screening, eating habits, and promotion of physical activity) were also assessed using Student’s t-tests and ANOVA.

Multiple linear regression was performed to identify the independent factors that are associated with the knowledge score. Variables that showed a statistically significant association (*p* < 0.10) in the bivariate analysis were included in the multivariate model. Unstandardized coefficients (B), their 95% confidence intervals (95% CI), and *p*-values were reported. Multicollinearity was assessed using variance inflation factors (VIFs) and tolerance values. Statistical significance for all other analyses was defined at the 5% level (*p* < 0.05).

The collected data were analyzed using SPSS statistical software (version 26.0).

### Ethical approval and consent to participate

The study was approved by the Institutional Review Board (IRB) of our university (University Ethics Center of Université Saint-Joseph de Beyrouth (Reference: USJ-2024-201)). All procedures involving human participants were conducted in accordance with relevant guidelines and regulations, including the ethical principles of the Declaration of Helsinki. Participation was voluntary, and completion of the anonymous questionnaire was considered to imply informed consent and was approved by the IRB; therefore, the IRB waived the requirement for separate written informed consent.

## Results

### Sociodemographic characteristics of the population

A total of 946 parents were included in the analysis. The mean age of participants was 43.9 ± 6.2 years, ranging from 25 to 68 years. The population was predominantly female (91.0%). The level of education was high, with 94.1% of parents having a university degree. In terms of professional occupation, 11.6% were healthcare professionals. Most parents had two children (50.5%), followed by those with three children (27.2%) (Table 1).

**Table 1:** Sociodemographic characteristics of the study population

		*N* = 946 *n* (%)
Age (years)	M ± SD	43.9 ± 6.2
	Min – Max	25–68
Sex	Male	85 (9.0%)
	Female	861 (91.0%)
Level of education	Primary school	15 (1.6%)
	High school	41 (4.3%)
	University	890 (94.1%)
Professional Occupation	Healthcare professional	110 (11.6%)
	Other	836 (88.4%)
Number of children	1	154 (16.3%)
	2	478 (50.5%)
	3	257 (27.2%)
	4	51 (5.4%)
	5	6 (0.6%)

### Parental awareness of pediatric dyslipidemia

Among the 946 parents surveyed, less than half (42.4%, *n* = 401) reported having heard of dyslipidemia in children, while 57.6% reported never having heard of it. Among parents who were aware of this problem, the main source of information was the media (television, internet, and other media), cited by 25.2% (*n* = 101) of respondents, followed by healthcare professionals (24.4%, *n* = 98), other sources (22.2%, *n* = 89), friends (17.5%, *n* = 70), family (7.5%, *n* = 30), and finally school (3.2%, *n* = 13). Regarding self-assessed knowledge, most participants reported limited awareness of pediatric dyslipidemia: 44.7% (*n* = 423) indicated no knowledge, and 32.9% (*n* = 311) reported little knowledge, while only 15.8% (*n* = 149) rated their knowledge as average, and 6.7% (*n* = 63) as very good.

### Parents’ knowledge score

**Fig. 1:**
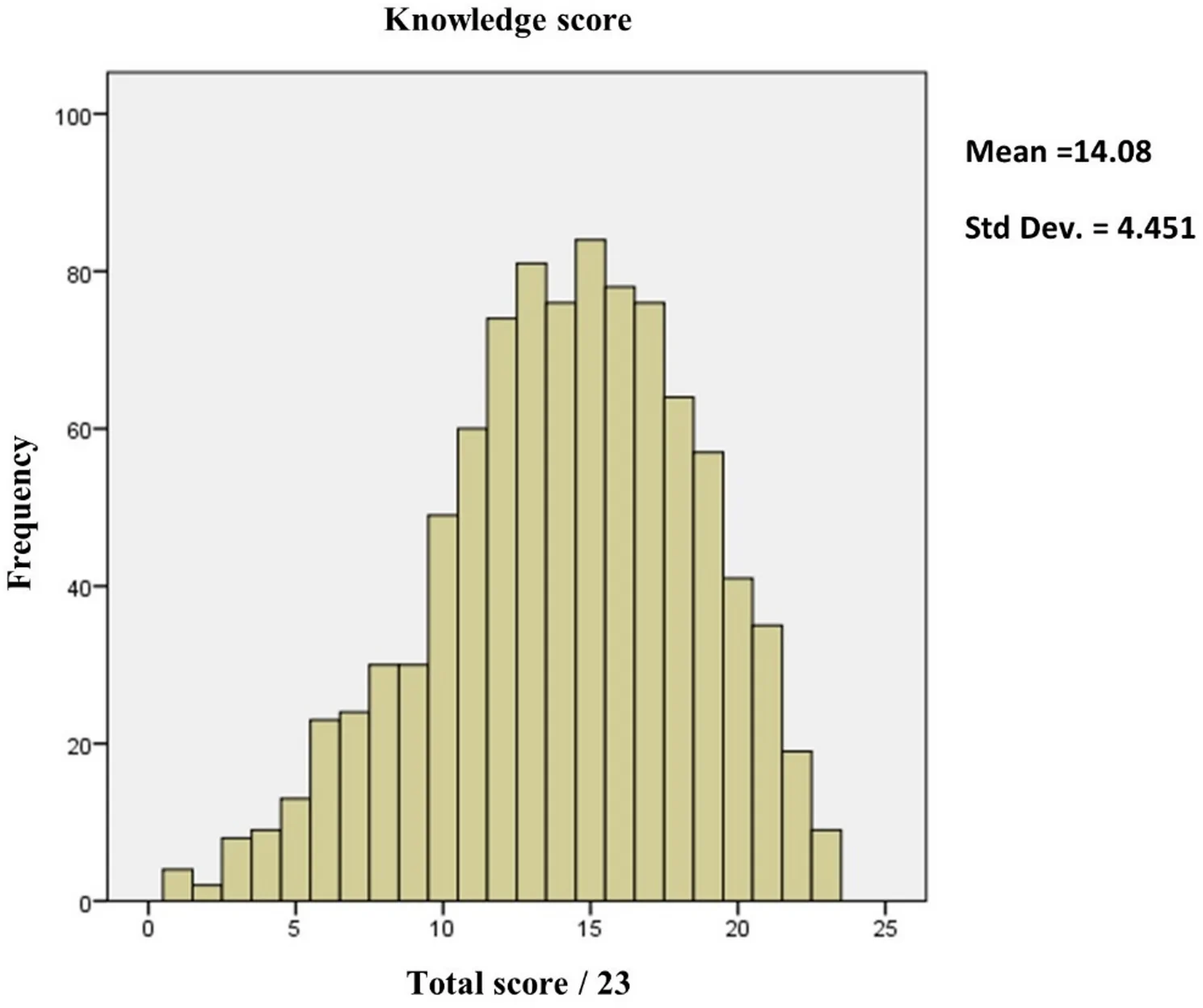
Knowledge scores within the study population

Only about half of parents (50.5%) believed that their children should be screened for dyslipidemia, and 45.8% answered the question about the age at which screening should begin correctly. An overall knowledge score out of 23 points was established to assess parents’ level of knowledge, with the questions and correct answers presented in Table 2. The mean knowledge score was 14.1 ± 4.4 out of 23, with a median of 14.0 [11.0–17.0] and scores ranged from 1 to 23(Fig. 1).

**Table 2:** Parents’ knowledge of pediatric dyslipidemia

		*n* (%)
According to current guidelines, should children be screened for dyslipidemia?	No	468 (49.5%)
	Yes *	478 (50.5%)
At what age do you think screening for dyslipidemia should begin?	9 to 11 years *	433 (45.8%)
	21 years and older	150 (15.9%)
	30 years and older	55 (5.8%)
	Only in case of medical problems	308 (32.6%)
In your opinion, which of the following risk factors are associated with dyslipidemia in children?	Family history of heart disease *	690 (72.9%)
	High-fat diet *	728 (77.0%)
	Lack of physical activity *	643 (68.0%)
	Obesity *	687 (72.6%)
	High sugar intake *	617 (65.2%)
To your knowledge, what diseases can be caused by dyslipidemia?	Myocardial infarction (Heart attack) *	537 (56.8%)
	Atherosclerosis (Fat buildup in blood vessels) *	381 (40.3%)
	Hepatic steatosis (fatty liver) *	557 (58.9%)
	Diabetes *	583 (61.6%)
	Acute pancreatitis (inflammation of the pancreas) *	280 (29.6%)
How do you think dyslipidemia is diagnosed?	By a blood test *	780 (82.5%)
	By physical examination	7 (0.7%)
	By genetic testing	18 (1.9%)
	By diet evaluation	12 (1.3%)
	I don’t know	129 (13.6%)
In your opinion, what are the management strategies for dyslipidemia in children that you are familiar with?	Diet changes *	750 (79.3%)
	Increased physical activity *	691 (73.0%)
	Medication *	267 (28.2%)
	Regular lipid profile monitoring *	373 (39.4%)
	Lifestyle advice *	509 (53.8%)
	I don’t know	116 (12.3%)
In your opinion, what preventive measures can help reduce the risk of dyslipidemia in children?	Healthy eating habits *	894 (94.5%)
	Regular physical exercise *	831 (87.8%)
	Limiting screen time *	356 (37.6%)
	Regular health checkups *	452 (47.8%)
	Health education for children *	617 (65.2%)

Correct answers (*)

### Parental practices

Blood test-based lipid screening for dyslipidemia in children had been performed by only 6.7% of parents. Among those who had been screened, results were reported as normal in 44.4% of cases, high in 14.3% of cases, and borderline in 7.9% of cases, while 33.3% of parents reported not knowing the results, and only 4.8% received treatment for dyslipidemia (Table 3).

**Table 3:** Parental practices and preventive attitudes regarding pediatric dyslipidemia

		*n* (%)
Have you ever screened your children for dyslipidemia using a blood test? Yes		63 (6.7%)
If yes, lipid profile results*	Unknown	21 (33.3%)
	Normal	28 (44.4%)
	Borderline	5 (7.9%)
	High	9 (14.3%)
Do your children receive treatment for dyslipidemia? Yes		3/63 (4.8%)
Do you encourage your children to be physically active?		938 (99.2%)
If yes, how often?	Daily	102 (10.9%)
	Once a week	104 (11.1%)
	Twice a week	376 (40.1%)
	Three times or more	356 (38.0%)
Do you provide your children with a balanced diet?	Never	6 (0.6%)
	Rarely	9 (1.0%)
	Sometimes	311 (32.9%)
	Always	620 (65.5%)
Do you provide your children with vegetables and fruits?	Every day	810 (85.6%)
	Twice a week	104 (11.0%)
	Once a week	29 (3.1%)
	Never	3 (0.3%)
Would you be interested in further information about dyslipidemia in children? Yes		832 (87.9%)
What type of resources would be most useful to you?	Information brochures	259 (27.4%)
	Workshops or seminars	163 (17.2%)
	Online resources	546 (57.7%)
	Individual consultations with health professionals	181 (19.1%)
	School programs	454 (48.0%)

*Results provided by parents were categorized into normal, borderline or high according to the NHLBI [8]

Nearly all parents (99.2%) reported encouraging their children to engage in physical activity. In addition, 65.5% reported that they were always providing their children with a balanced diet, while 32.9% reported doing so sometimes. Fruit and vegetable consumption was reported as daily in 85.6% of children. Finally, a large majority of parents (87.9%) expressed an interest in receiving additional information on pediatric dyslipidemia. Online resources were the most frequently cited type of support (57.7%), followed by school programs (48.0%) and information brochures (27.4%) (Table 3).

### Factors affecting knowledge scores

**Fig. 2:**
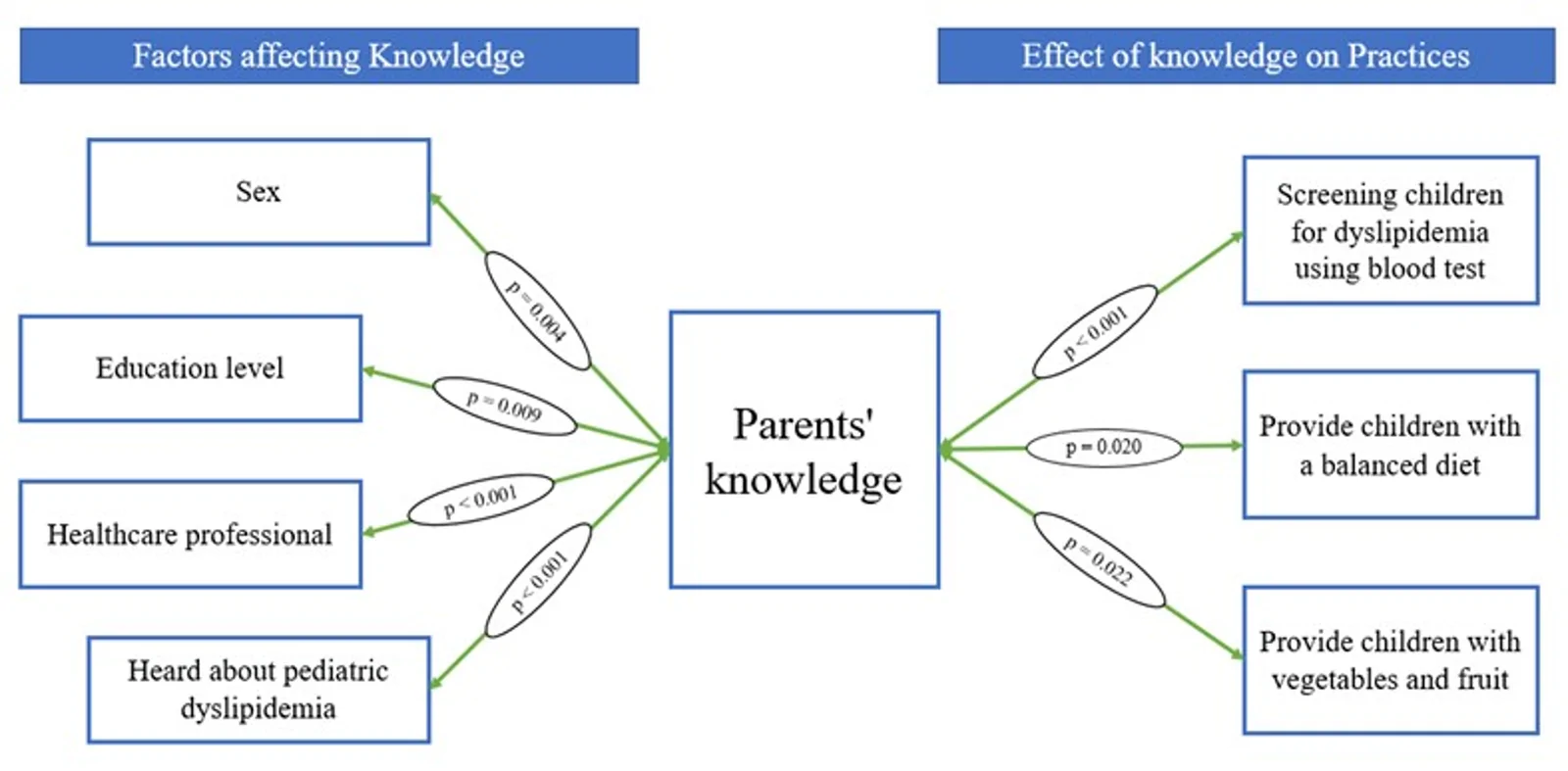
Parental knowledge: influencing factors and effect on preventive practices

Bivariate analysis showed that female gender, university education, and being a healthcare professional were associated with a higher knowledge score (respectively: 14.2 ± 4.4 vs. 12.4 ± 4.8; *p* < 0.001 for female gender; 14.2 ± 4.4 vs. 12.5 ± 4.8; *p* = 0.007 for university education; and 17.3 ± 3.6 vs. 13.7 ± 4.4; *p* < 0.001 for being a healthcare professional). Similarly, parents who had heard of dyslipidemia in children had a significantly higher knowledge score than those who had never heard of it (15.8 ± 3.9 vs. 12.8 ± 4.4; *p* < 0.001). However, the parents’ age and the number of children were not correlated with the knowledge score (*p* = 0.531 and *p* = 0.221, respectively) (Table 4; Fig. 2).

**Table 4:** Factors associated with parents’ knowledge score

		M ± SD	*P*.value
Sex	Male	12.4 ± 4.8	**< 0.001**
	Female	14.2 ± 4.4	
Level of education	Non-university	12.5 ± 4.8	**0.007**
	University	14.2 ± 4.4	
Healthcare professional	No	13.7 ± 4.4	**< 0.001**
	Yes	17.3 ± 3.6	
Have you heard of dyslipidemia in children?	No	12.8 ± 4.4	**< 0.001**
	Yes	15.8 ± 3.9	
Age	*r*	-0.020	0.531
Number of children	1 child	13.9 ± 4.9	0.221
	2 children	14.3 ± 4.3	
	3 children and more	13.8 ± 4.4	

*M ± SD *Mean ± Standard deviation, *r *Pearson Correlation coefficient

In the Multiple linear regression (Table 5), the regression model explained 16.1% of the variance for the knowledge score (*R*² = 0.161; adjusted R² = 0.158). Female gender, university education level, and working in a healthcare profession were independently associated with the knowledge score, with an average increase in the score of 1.35 points (B = 1.35; 95% CI [0.430–2.269]; *p* = 0.004) for female gender, 1.48 points (B = 1.478; 95% CI [0.363–2.593]; *p* = 0.009) for university education, and 2.32 points (B = 2.324; 95% CI [1.472–3.176]; *p* < 0.001) for being a healthcare professional. In addition, parents who had heard of dyslipidemia in children had a significantly higher knowledge score, with an average increase of 2.65 points (B = 2.653; 95% CI [2.102–3.204]; *p* < 0.001).

**Table 5:** Multiple linear regression examining the independent factors affecting the knowledge score (*R*² = 0.161; adjusted *R*² = 0.158)

Model	Unstandardized Coefficients B	*P*.value	95.0% Confidence Interval for B		Collinearity Statistics	
			Lower Bound	Upper Bound	Tolerance	VIF
Sex	1.350	0.004	0.430	2.269	0.983	1.018
University education level	1.478	0.009	0.363	2.593	0.982	1.019
Healthcare professional	2.324	< 0.001	1.472	3.176	0.910	1.099
Parents who have already heard about pediatric dyslipidemia	2.653	< 0.001	2.102	3.204	0.917	1.091

*Dependent Variable: Knowledge Score*

### Association between the knowledge score and preventive practices

The bivariate analysis revealed a significant association between parents’ level of knowledge and several preventive practices related to pediatric dyslipidemia (Table 6; Fig. 2). Parents who had already screened their children for dyslipidemia had a significantly higher knowledge score than those who had never performed this screening (16.9 ± 3.5 vs. 13.9 ± 4.4; *p* < 0.001). Parents who reported always providing a balanced diet for their children had a higher knowledge score (14.4 ± 4.4) compared to those who reported doing so sometimes (13.5 ± 4.5), rarely (14.0 ± 5.5), or never (12.5 ± 6.1) (*p* = 0.02). Similarly, parents who reported that their children consumed fruit and vegetables every day had a higher knowledge score (14.3 ± 4.4) than those who reported less frequent consumption, particularly once (13.0 ± 4.3) or twice (13.0 ± 4.8) a week, or never (11.7 ± 5.5) (*p* = 0.02).

**Table 6:** Association between knowledge score and preventive practices

		M ± SD	*P*.value
Have you ever screened your children for dyslipidemia using blood test?	No	13.9 ± 4.4	**< 0.001**
	Yes	16.9 ± 3.5	
Do you provide your children with a balanced diet?	Never	12.5 ± 6.1	**0.020**
	Rarely	14.0 ± 5.5	
	Sometimes	13.5 ± 4.5	
	Always	14.4 ± 4.4	
Do you provide your children with vegetables and fruit?	Every day	14.3 ± 4.4	**0.022**
	Twice a week	13.0 ± 4.8	
	Once a week	13.0 ± 4.3	
	Never	11.7 ± 5.5	

*M ± SD *Mean ± Standard deviation

## Discussion

In our study, we assessed the knowledge and practices of a large sample of Lebanese parents regarding dyslipidemia in children and adolescents. A total of 946 parents of students attending eight Lebanese schools agreed to participate in the study, with most of them being female (91%). Our findings indicate that, although 94% of the study population had attained a university degree, overall parental awareness of pediatric dyslipidemia remains limited. In fact, only 42.4% of parents had heard of this condition, and only half of them considered screening their children necessary. Moreover, the mean knowledge score on pediatric dyslipidemia was only 14.1 out of 23. Finally, only 6.7% of parents reported that their children had undergone blood test-based lipid screening.

To date, relatively few studies have addressed this topic on a global scale. A previous US study involving 91 parents similarly found that 45.5% supported universal screening for dyslipidemia [19]. The same study further reported that over 80% of parents recognized the association between nutrition, physical activity, genetic factors, and pediatric dyslipidemia [19]. Parents’ limited knowledge of pediatric dyslipidemia may be partly explained by a lack of awareness of dyslipidemia among adults, as several studies indicate that the majority of affected adults remain unaware of their condition [14, 15, 17]. In fact, the percentage of people aware of their disease was reported at 21% in the Bogalusa study [14], only 15% in the ORISCAV-LUX Luxembourg study [15], and 14.4% in a Chinese study [17].

Multivariate analysis showed that female gender, university education level, healthcare professional status, and having heard of pediatric dyslipidemia were independent determinants of higher parental knowledge score. 91% of participants in the present study were women, with significantly higher knowledge scores than men. This high female response rate is comparable to that reported in the study by Kern et al. [19], in which 81.3% of respondents were women. The observed predominance of female responses may be explained by mothers’ greater involvement in their children’s health and care. They are the ones who take their children to the pediatrician, are more involved in school-related communication, and are more present in parent communication groups. Accordingly, mothers often constitute the main point of interaction between the school and the child, as they are more likely to receive and complete the school’s questionnaires, whereas fathers may be less involved in these communication processes.

The parents’ level of university education was associated with a higher knowledge score, whereas parental age was not. To our knowledge, no previous studies have examined the association between parental age or education level and knowledge of pediatric dyslipidemia. However, in adults, age and a high level of education were associated with better knowledge of their dyslipidemia status [14, 17]. These findings highlight the strong link between educational attainment and dyslipidemia knowledge in both populations.

The findings of this study also showed that being a healthcare professional parent was associated with higher knowledge scores. These results are consistent with those observed in the Korean adult population, which show that healthcare professionals had a better understanding of adult dyslipidemia [16]. This finding can be attributed to greater exposure to the latest evidence-based medical guidelines on pediatric dyslipidemia.

Despite the very high prevalence of pediatric dyslipidemia, screening in children remains insufficient. The low screening rate (6.7%) observed in our study is comparable to those reported before the publication of the 2011 NHLBI recommendations. Indeed, studies conducted between 1995 and 2012 reported that only 3.4% [22] to 16% [23] of children were screened for dyslipidemia. More recently in the US, screening rates have increased modestly, with 9.0% of 9- to 11-year-olds and 12.9% of 17- to 21-year-olds undergoing lipid screening; however, overall rates remain low [24]. Although lipid screening appears to have increased in more recent years, with nearly one-third of young people aged 16 to 21 having undergone screening [25], a very recent study reported a decline in cholesterol screening following the COVID-19 pandemic and the updated U.S. Preventive Services Task Force recommendations [26].

To improve screening, several studies have evaluated the impact of educational interventions targeting healthcare professionals. In one study, following an intervention, the proportion of patients for whom screening tests were prescribed increased from 8.9% to 50% [27], while in another study, it increased from 6.2% to 84.8% [28]. These findings highlight the potential effectiveness of such interventions in supporting clinicians’ adherence to recommendations. However, the actual performance of the prescribed tests remained limited. In fact, only 69.6% of children actually underwent screening in the first study [27] and 56.7% in the second [28]. Thus, even when physicians adhere to recommendations, overall screening rates remain insufficient. This suggests that parental barriers may contribute to the gap between prescription and actual screening. Despite the 2011 recommendations, 30.8% of parents still disagreed with universal screening for children [19]. More recently, Scully P. et al. reported that 98% of parents supported screening for their children [20]. However, this study was based on a small sample of 53 parents of hospitalized children and may not be representative of the general population.

The exceptionally high rates of favorable practices reported by parents, such as 99.2% of respondents claiming to encourage physical activity and 85.6% reporting daily fruit and vegetable consumption, can be explained by a possible desirability bias. Parents may have over-reported these healthy behaviors to conform to perceived social norms or medical expectations regarding ideal parenting practices. However, our study lacked objective behavioral verification such as dietary logs, physical activity trackers, or direct clinical observations. Therefore, these high percentages should be interpreted with caution.

Our findings of low screening rates should be interpreted in the context of the ongoing international debate regarding pediatric lipid screening strategies. Although our data suggest a significant gap in screening implementation, determining whether Lebanon should adopt a universal or strictly targeted screening framework requires careful consideration of these differing international perspectives.

We examined the association between parents’ knowledge scores and the implementation of favorable preventive practices. Higher parental scores were significantly associated with more favorable preventive practices, including lipid screening and healthier eating habits in children. These findings highlight the importance of improving knowledge as it may contribute to the development of better preventive practices. By adopting healthier preventive practices, parents may help improve their children’s lipid profiles and reduce the risk of future metabolic diseases such as obesity and diabetes. In addition, screening according to the AAP recommended guidelines may facilitate early diagnosis and management before the development of atherosclerosis and cardiovascular disease.

Finally, among parents who had heard of dyslipidemia, only 24.4% reported obtaining information from a healthcare professional. This finding can be explained by the fact that only 26% of pediatricians are aware of the current screening recommendations [29]. Hence, strengthening healthcare professionals’ education is essential to raise awareness among parents and increase screening rates in children. Indeed, in a US study conducted after the implementation of educational and awareness-raising measures for pediatricians on the new recommendations, the screening rate increased from 8.9% to 50% in 12 months [27].

As a next step, we intend to organize awareness sessions and distribute digital materials to parents, students, and teachers in schools. These educational sessions aim to meet the informational needs reported by nearly 90% of surveyed parents, who expressed a desire for further guidance on pediatric and adult dyslipidemia, through online resources, school-based programs, or informational brochures. These educational materials should address pediatric dyslipidemia, including its definition, causes, and consequences, as well as recommended prevention and management strategies. Subsequently, an online knowledge evaluation will be conducted to measure the impact of these educational interventions on participants’ knowledge scores.

The main strengths and limitations of the present study are summarized in Table 7. Regarding limitations, first, the data were based on a self-reported questionnaire, which may be subject to social desirability, potentially leading to overestimation of knowledge and appropriate practice. Second, the study recruited parents exclusively from private schools in Beirut and the Mount Lebanon region using a convenience sampling strategy. Public schools were not included. This may have resulted in the overrepresentation of urban, socioeconomically advantaged, and highly educated parents, as reflected by the high proportion of respondents with university education. The approximate 25% response rate may also have introduced selection bias, as parents who chose to participate may have been more interested in child health, prevention, or dyslipidemia than non-respondents. Moreover, because the questionnaire was distributed electronically and participation was voluntary, responses may have been more likely to come from parents with higher levels of education or greater access to digital tools. Another limitation is that the questionnaire did not collect information on parental history of dyslipidemia, the respondent’s relationship to the child, prior formal education about dyslipidemia, or the presence of chronic metabolic diseases among children. The absence of these factors may therefore have limited our ability to account for other potential confounding factors, as these variables could influence parental knowledge and preventive practices. Finally, because of the cross-sectional design, causal relationships between knowledge, attitudes, and practices could not be established.

**Table 7:** Summary of the main strengths and limitations of the study

Main strengths	Main limitations
Large, multilingual sample recruited from several schools.	Convenience sampling from private schools only, limiting generalizability.
Questionnaire developed from the literature, reviewed by experts, and pilot-tested.	Self-reported online data, social desirability, and selection biases.
Composite knowledge score with good internal consistency.	Limited formal psychometric validation.
First study providing data on parental knowledge and practices regarding pediatric dyslipidemia in Lebanon.	Cross-sectional design and missing potential confounding variables.

Although the questionnaire was reviewed by pediatric experts and pilot-tested among parents before data collection, formal psychometric validation was limited. A Content Validity Index was not calculated, and test-retest reliability was not assessed due to the anonymous cross-sectional design. Therefore, further validation studies are needed before using this instrument in longitudinal research or to evaluate the impact of educational interventions.

Despite these limitations, the present study has several strengths, including its relatively large sample size, recruitment from several schools, and good internal consistency among the knowledge score items. In addition, the questionnaire was designed and distributed in three languages to maximize response rates and ensure accessibility to parents from different socioeconomic backgrounds. Furthermore, parental knowledge related to pediatric dyslipidemia was assessed for the first time using a composite score covering several domains, including screening indications, risk factors, and strategies for managing dyslipidemia in children. This scoring system may provide a reliable quantitative assessment of knowledge, allowing longitudinal tracking of changes in knowledge following educational intervention.

These findings highlight the need for coordinated preventive strategies involving schools, healthcare professionals, and public health authorities. Schools may play an important role in disseminating health information to parents and promoting healthy lifestyle behaviors among children. Pediatricians should also be supported through continuing education to ensure consistent counseling and screening practices. At the national level, clearer guidance on pediatric dyslipidemia screening in Lebanon could help reduce variability in physicians’ screening practices and improve early detection. In addition, digital health campaigns through school platforms, parent communication groups, and social media may represent accessible tools to improve parental awareness and engagement.

## Conclusion

In conclusion, while universal lipid screening remains a subject of ongoing international debate, this study highlights insufficient parental knowledge of pediatric dyslipidemia and a low rate of screening among children. Strengthening parental education and preventive counseling may help promote early screening and reduce future cardiovascular risk.

The development of point-of-care testing (POCT), which measures cholesterol and triglyceride levels from a single drop of capillary blood, is likely to facilitate more widespread screening for dyslipidemia in children by enhancing parental acceptance. In fact, previous research demonstrated that the use of POCT has improved the diagnosis of hypercholesterolemia in children, due to its many advantages: ease of use, speed, portability, low cost, and fewer visits to the laboratory [30].

## Supplementary Information

Supplementary Material 1.

Supplementary Material 2.

Supplementary Material 3.

## Data Availability

Data are available upon request from the corresponding author.

## References

[1] Lipid Disorders in Children and Adolescents. In: Sperling Pediatric Endocrinology. Elsevier. 2021. pp. 1004–21. Available from: https://www.sciencedirect.com:5037/science/chapter/edited-volume/abs/pii/B9780323625203000257. [Cited 14 Dec 2025].

[2] Wiegman A, Gidding SS, Watts GF, Chapman MJ, Ginsberg HN, Cuchel M, et al. Familial hypercholesterolaemia in children and adolescents: gaining decades of life by optimizing detection and treatment. Eur Heart J. 2015;36(36):2425–37. 10.1093/eurheartj/ehv157. PMC4576143 26009596

[3] Mosca S, Araújo G, Costa V, Correia J, Bandeira A, Martins E, et al. Dyslipidemia Diagnosis and Treatment: Risk Stratification in Children and Adolescents. J Nutr Metabolism. 2022;2022(1):4782344. 10.1155/2022/4782344. PMC8885266 35237450

[4] Kit BK, Kuklina E, Carroll MD, Ostchega Y, Freedman DS, Ogden CL. Prevalence of and Trends in Dyslipidemia and Blood Pressure Among US Children and Adolescents, 1999–2012. JAMA Pediatr. 2015;169(3):272–9. 10.1001/jamapediatrics.2014.3216. PMC7423159 25599372

[5] Perak AM, Ning H, Kit BK, de Ferranti SD, Van Horn LV, Wilkins JT, et al. Trends in Levels of Lipids and Apolipoprotein B in US Youths Aged 6 to 19 Years, 1999–2016. JAMA. 2019;321(19):1895–905. 10.1001/jama.2019.4984. PMC6537842 31112258

[6] Gannagé-Yared MH, Farah V, Chahine E, Balech N, Ibrahim T, Asmar N, et al. Distribution and correlates of non–high-density lipoprotein cholesterol and triglycerides in Lebanese school children. J Clin Lipidol. 2016;10(2):378–85. 12.018 PubMed PMID: 27055969. 10.1016/j.jacl.2015.12.018 27055969

[7] Georges N, Simon A, Naim B, Georges N, Georges AF, Tanios A. Universal screening program for lipid disorders in 2–10 years old Lebanese children: A new approach. Int J Pediatr Adolesc Med. 2019;6(3):101. 10.1016/j.ijpam.2019.05.003. PMC6824154 31700968

[8] Expert panel on integrated guidelines for cardiovascular health and risk reduction in, children and adolescents. Expert Panel on Integrated Guidelines for Cardiovascular Health and Risk Reduction in Children and Adolescents: Summary Report. Pediatrics. 2011;128(Supplement5):S213–56. 10.1542/peds.2009-2107C. PMC4536582 22084329

[9] Srinivasan SR, Frontini MG, Xu J, Berenson GS. Utility of Childhood Non–High-Density Lipoprotein Cholesterol Levels in Predicting Adult Dyslipidemia and Other Cardiovascular Risks: The Bogalusa Heart Study. Pediatrics. 2006;118(1):201–6. 10.1542/peds.2005-1856. 16818566

[10] Pires A, Sena C, Seiça R. Dyslipidemia and cardiovascular changes in children. Curr Opin Cardiol. 2016;31(1):95. 10.1097/HCO.0000000000000249. 26599060

[11] Dwyer T, Jacobs DR, Woo JG, Urbina EM, Bazzano L, Juonala M, et al. 1447Childhood risk factors and cardiovascular disease outcomes in adulthood. Preliminary findings from the International Childhood Cardiovascular Cohort (i3C) Consortium. Eur Heart J. 2019;40(Supplement1):ehz7480082. 10.1093/eurheartj/ehz748.0082.

[12] US Preventive Services Task Force. Screening for Lipid Disorders in Children and Adolescents: US Preventive Services Task Force Recommendation Statement. JAMA. 2023;330(3):253–60. 10.1001/jama.2023.11330. 37462699

[13] Patro-Golab B, Le Thi TG, Litwin AM, Grünzner J, Koletzko B. Current guidelines on screening, diagnosis and management of dyslipidaemia in childhood: a systematic review. Pediatr Res. 2025;1–8. 10.1038/s41390-025-04572-2. 41238902

[14] Frontini MG, Srinivasan SR, Elkasabany A, Berenson GS. Awareness of hypertension and dyslipidemia in a semirural population of young adults: the bogalusa heart study. Prev Med. 2003;36(4):398–402. 10.1016/S0091-7435(02)00045-2. 12649047

[15] Alkerwi A, Pagny S, Lair ML, Delagardelle C, Beissel J. Level of Unawareness and Management of Diabetes, Hypertension, and Dyslipidemia among Adults in Luxembourg: Findings from ORISCAV-LUX Study. PLoS ONE. 2013;8(3):e57920. 10.1371/journal.pone.0057920. PMC3587422 23483942

[16] Bae JH, Jin ES, Kim SE, Kang S, Lee JY, Kim M, et al. Public Awareness of Dyslipidemia Among the Korean Population: A Survey Study. J Lipid Atherosclerosis. 2023;12(3):307–14. 10.12997/jla.2023.12.3.307. PMC10548193 37800113

[17] Zhang FL, Xing YQ, Wu YH, Liu HY, Luo Y, Sun MS, et al. The prevalence, awareness, treatment, and control of dyslipidemia in northeast China: a population-based cross-sectional survey. Lipids Health Dis. 2017;16(1):61. 10.1186/s12944-017-0453-2. PMC5363017 28330492

[18] Alahmari AS, Alrashed FM, Awad AR, Khozam SA, Alshahrani MM, Mahdi BA , et al. Knowledge, attitude and practice towards dyslipidemia among patients attending primary health care centers, Abha City, Saudi Arabia. International Journal of Advanced Research. 2016;4(11):1937–46. 10.21474/IJAR01/2296.

[19] Kern L, Eichberger L, Wang H, Lin T, Rhee KE. Parental Knowledge and Attitudes About Universal Lipid Screening Among Children Aged 9 to 11 Years. Clin Pediatr (Phila). 2020;59(4–5):439–44. 10.1177/0009922820903043. PMC9653267 32003230

[20] Scully P, Waldron J, Deeny H, King K, O’Neill MB, Macken AP, et al. Feasibility and Parental Attitudes to Universal Cholesterol Screening in Paediatric in-Patients. Ir Med J. 2021;114(1):237. PubMed PMID: 37555921. 37555921

[21] Brislin RW. Back-Translation for Cross-Cultural Research. J Cross-Cult Psychol. 1970;1(3):185–216. 10.1177/135910457000100301.

[22] Vinci SR, Rifas-Shiman SL, Cheng JK, Mannix RC, Gillman MW, de Ferranti SD. Cholesterol Testing Among Children and Adolescents During Health Visits. JAMA. 2014;311(17):1804–7. 10.1001/jama.2014.2410. 24794376

[23] Zachariah JP, McNeal CJ, Copeland LA, Fang-Hollingsworth Y, Stock EM, Sun F, et al. Temporal trends in lipid screening and therapy among youth from 2002 to 2012. J Clin Lipidol. 2015;9(5 Suppl):S77–87. 10.1016/j.jacl.2015.06.014 . PubMed PMID: 26343215; PubMed Central PMCID: PMC4562073. PMC4562073 26343215

[24] Thompson-Paul AM, Kraus EM, Porter RM, Pierce SL, Kompaniyets L, Sekkarie A, et al. Pediatric Lipid Screening Prevalence Using Nationwide Electronic Medical Records. JAMA Netw Open. 2024;7(7):e2421724. 10.1001/jamanetworkopen.2024.21724. PMC11267408 39042409

[25] Li N, Zhang J, Huang Y, Wang S, Gao X, Yang Z, et al. Prevalence of and factors associated with lipid screening in young people aged 16– to 21 years in the United States: analysis of nationwide cross-sectional data. Lipids Health Dis. 2024;23(1):350. 10.1186/s12944-024-02336-1. PMC11514873 39462389

[26] Schwartz JK, Zhang X, Peterson AL. Changes in Youth Cholesterol Screening Rates in an Academic Center During the COVID-19 Pandemic. Pediatr Cardiol. 2026;47(2):752–8. 10.1007/s00246-025-03831-7. 40116884

[27] DeSantes K, Dodge A, Eickhoff J, Peterson AL. Improving Universal Pediatric Lipid Screening. J Pediatr. 2017;188:87–90. 10.1016/j.jpeds.2017.05.030 . PubMed PMID: 28595766. 28595766

[28] Kern L, Crow J, Williams CB, Boies E, Gahagan S, Rhee KE. Increasing Universal Lipid Screening Among 9- to 11-Year-Old Children Through a Quality Improvement Initiative. Clin Pediatr (Phila). 2017;56(7):640–7. 10.1177/0009922816670979. 27663965

[29] de Ferranti SD, Rodday AM, Parsons SK, Cull WL, O’Connor KG, Daniels SR, et al. Cholesterol Screening and Treatment Practices and Preferences: A Survey of United States Pediatricians. J Pediatr. 2017;185:99–e1052. 10.1016/j.jpeds.2016.12.078 . PubMed PMID: 28209292. 28209292

[30] Arriaga-Cázares HE, Vega-Morales D, Moreno-Treviño CA, Juarez-Juarez JL, Pérez-Arizmendi CA, Martagón-Rosado AJ. Comparison of Two Strategies for Hypercholesterolemia Detection through Point-of-Care Testing. Diagnostics. 2024;14(2):143. 10.3390/diagnostics14020143. PMC10814133 38248020

