# Supplementary material for: Dyslipidemia in Lebanese children and adolescents: parents’ knowledge and practices
Source: BMC Public Health. 2026 Jun 24;26:2801. doi: 10.1186/s12889-026-28254-8 (PMC13637342; doi:10.1186/s12889-026-28254-8)
Supplement: Supplementary file 1 [file 12889_2026_28254_MOESM1_ESM.pdf]

Comité d'éthique de la recherche
Université Saint-Joseph
APPROUVÉ

Information sheet

Dear Sir/Madam,

My name is Khalil Hamadeh, and I am a 7th-year student at the Faculty of Medicine, Saint Joseph University (USJ). I am working on my medical thesis under the supervision of Pr Marie-Hélène Gannagé-Yared (e-mail: mariehelene.yared@usj.edu.lb, tel.: 03 291301).

I would like to invite you to participate in a research project entitled "Pediatric Dyslipidemia in Lebanon: Knowledge and Practices of Parents," which examines parents' knowledge and practices regarding this health issue in children.

This project has been approved by the Ethics Committee of Saint Joseph University (Ref. USJ-2024-201).

Dyslipidemia is defined as an abnormal level of lipids (cholesterol, triglycerides, HDL-Cholesterol). It is common in children and adolescents and has become a public health problem in recent years. The number of children and adolescents diagnosed with this condition is increasing. In Lebanon, the incidence is particularly high, affecting up to one in 4 children. Dyslipidemia in Lebanese children is very common, with all the negative long-term consequences it can have on their health. Early detection of this disease can help identify children at risk and prevent cardiovascular disease in adulthood.

Your participation in this survey is **voluntary**. By participating in this study, you will be doing us a great service and enabling us to learn more about the management of pediatric dyslipidemia in Lebanon and parents' attitudes towards this subject. Your participation would be invaluable in improving our understanding and promoting healthier lifestyles.

This questionnaire respects your **anonymity,** and the content of your responses will be treated with the strictest **confidentiality**.

You are free to answer any or all questions.

Thank you in advance for your time.

For further information, please contact Khalil Hamadeh (e-mail: khalil.hamadeh@net.usj.edu.lb, tel. 71 210807) or my thesis supervisor, Pr. Marie-Hélène Gannagé-Yared (e-mail: mariehelene.yared@usj.edu.lb, tel. 03 291301).

Comité d'éthique de la recherche
Université Saint-Joseph
APPROUVÉ

Questionnaire

Part 1. Sociodemographic and personal characteristics

1. Age :

2. Sex: Male/Female

3. Level of education:
   a) Primary school
   b) High school
   c) University

4. Professional status:
   a) Healthcare professional
   b) Other

5. How many children do you have?

Part 2: Parental awareness of pediatric dyslipidemia

1. Have you heard of dyslipidemia (lipid abnormalities) in children?
   a) Yes
   b) No

2. If yes, how did you first hear about this problem?
   a) From a health professional (doctor, nurse...)
   b) At school
   c) From the media (TV, Internet, etc.)
   d) From family or friends
   e) Other (please specify): ________________

3. How would you describe your current level of knowledge about dyslipidemia in children?
   a) Very good knowledge
   b) Average knowledge
   c) Little knowledge
   d) No knowledge

Part 3: Parents' knowledge of pediatric dyslipidemia

1. Do you think that your children should be screened for dyslipidemia?
   a) Yes
   b) No

Comité d'éthique de la recherche
Université Saint-Joseph
APPROUVÉ

2. At what age do you think screening for dyslipidemia should begin?
   a) 9 to 11 years
   b) 21 and over
   c) 30 and over
   d) Only in case of medical problems

3. In your opinion, which of the following risk factors are associated with dyslipidemia in children? (Select all that apply)
   a) Family history of heart disease
   b) High-fat diet
   c) Lack of physical activity
   d) Obesity
   e) High sugar intake
   f) Other (please specify): ________________

4. To your knowledge, what diseases can be caused by dyslipidemia (Select all that apply)
   a) Myocardial infarction (Heart Attack)
   b) Atherosclerosis (Fat buildup in blood vessels)
   c) Hepatic steatosis (Fatty Liver)
   d) Diabetes
   e) Acute pancreatitis (inflammation of the pancreas)

5. How do you think dyslipidemia is diagnosed?
   a) By a blood test
   b) By physical examination
   c) By genetic testing
   d) By diet evaluation
   e) I don't know

6. In your opinion, what are the management strategies for dyslipidemia in children that you are familiar with? (Select all that apply)
   a) Diet changes
   b) Increased physical activity
   c) Medication
   d) Regular lipid profile monitoring
   e) Lifestyle advice
   f) Don't know
   g) Other (please specify): ________________

Comité d'éthique de la recherche
Université Saint-Joseph
APPROUVÉ

7. In your opinion, what preventive measures can help reduce the risk of dyslipidemia in children? (Select all that apply)
   a) I don't know
   b) Healthy eating habits
   b) Regular physical exercise
   c) Limiting screen time
   d) Regular health checkups
   e) Health education for children
   f) Other (please specify): ________________

Part 4. Screening practices and prevention habits

1. Have you ever screened your children for dyslipidemia using a blood test?
   a) Yes
   b) No

2. If yes, could you indicate the date and the results of the latest analysis of your children?

   Date:
   Results:
   - Cholesterol mg/dL or mmol/l
   - Triglycerides mg/dL or mmol/l
   - HDL-Cholesterol mg/dL or mmol/l

3. Do your children receive treatment for dyslipidemia?
   a) Yes
   b) No

4. Do you encourage your children to be physically active?
   a) Yes
   b) No

5. If yes, how often?
   a) Daily
   b) Once a week
   c) Twice a week
   d) Three times or more

6. Do you provide your children with a balanced diet?
   a) Never
   b) Rarely
   c) Sometimes
   d) Always

Comité d'éthique de la recherche
Université Saint-Joseph
APPROUVÉ

7. Do you provide your children with vegetables and fruits?
   a) Every day
   b) Twice a week
   c) Once a week
   d) Never

8. Would you be interested in further information about dyslipidemia in children?
   a) Yes
   b) No

9. What type of resources would be most useful to you? (Select all that apply)
   a) Information brochures
   b) Workshops or seminars
   c) Online resources
   d) Individual consultations with health professionals
   e) School programs
