# Supplementary material for: Dyslipidemia in Lebanese children and adolescents: parents’ knowledge and practices
Source: BMC Public Health. 2026 Jun 24;26:2801. doi: 10.1186/s12889-026-28254-8 (PMC13637342; doi:10.1186/s12889-026-28254-8)
Supplement: Supplementary file 2 [file 12889_2026_28254_MOESM2_ESM.pdf]

I. French version

Fiche d'information

Monsieur/Madame,

Je m'appelle Khalil Hamadeh et je suis étudiant en 7e année à la Faculté de Médecine de l'Université Saint-Joseph (USJ). Je réalise ma thèse de médecine sous la supervision du Pr Marie-Hélène Gannagé-Yared (e-mail : mariehelene.yared@usj.edu.lb, tél. : 03 291301).

Je souhaiterais vous inviter à participer à un projet de recherche intitulé *« Dyslipidémie pédiatrique au Liban : connaissances et pratiques des parents »*, qui examine les connaissances et les pratiques des parents concernant ce problème de santé chez les enfants.

Ce projet a été approuvé par le Comité d'éthique de l'Université Saint-Joseph (Réf. USJ-2024-201).

La dyslipidémie se définit comme un taux anormal de lipides (cholestérol, triglycérides, HDL-cholestérol). Elle est fréquente chez les enfants et les adolescents et constitue un problème de santé publique croissant. Le nombre d'enfants et d'adolescents diagnostiqués est en augmentation. Au Liban, l'incidence est particulièrement élevée, touchant jusqu'à un enfant sur quatre. La dyslipidémie chez les enfants libanais est très répandue, avec toutes les conséquences négatives à long terme qu'elle peut avoir sur leur santé. La détection précoce de cette maladie permet d'identifier les enfants à risque et de prévenir les maladies cardiovasculaires à l'âge adulte.

Votre participation à cette enquête est volontaire. En y prenant part, vous nous rendrez un grand service et nous permettrez d'en apprendre davantage sur la prise en charge de la dyslipidémie pédiatrique au Liban ainsi que sur les attitudes des parents à ce sujet. Votre contribution serait inestimable pour améliorer notre compréhension et promouvoir des modes de vie plus sains.

Ce questionnaire respecte votre anonymat et le contenu de vos réponses sera traité avec la plus stricte confidentialité.

Vous êtes libre de répondre à tout ou partie des questions. Nous vous remercions d'avance pour le temps que vous nous consacrerez.

Pour toute information complémentaire, veuillez contacter Khalil Hamadeh (e-mail : khalil.hamadeh@net.usj.edu.lb, tél. : 71 210807) ou mon directeur de thèse, Pr Marie-Hélène Gannagé-Yared (e-mail : mariehelene.yared@usj.edu.lb, tél. : 03 291301).

Questionnaire

Partie 1. Caractéristiques sociodémographiques et personnelles

1. Âge :

2. Sexe : Homme / Femme

3. Niveau d'éducation :

   a) École primaire
   b) École secondaire
   c) Université

4. Statut professionnel :

   a) Professionnel de santé
   b) Autre

5. Combien d'enfants avez-vous ?

Partie 2. Sensibilisation parentale à la dyslipidémie pédiatrique

1. Avez-vous entendu parler de la dyslipidémie (anomalies lipidiques) chez les enfants ?

   a) Oui
   b) Non

2. Si oui, comment avez-vous entendu parler de ce problème pour la première fois ?

   a) Par un professionnel de santé (médecin, infirmier…)
   b) À l'école
   c) Par les médias (TV, Internet, etc.)
   d) Par la famille ou des amis
   e) Autre (veuillez préciser) :

3. Comment décririez-vous votre niveau actuel de connaissance sur la dyslipidémie chez les enfants ?

   a) Très bonnes connaissances

b) Connaissances moyennes
c) Peu de connaissances
d) Aucune connaissance

Partie 3. Connaissances des parents sur la dyslipidémie pédiatrique

1. Pensez-vous que vos enfants devraient être dépistés pour la dyslipidémie ?

    a) Oui
    b) Non

2. Selon vous, à quel âge le dépistage de la dyslipidémie devrait-il commencer ?

    a) 9 à 11 ans
    b) 21 ans et plus
    c) 30 ans et plus
    d) Uniquement en cas de problèmes médicaux

3. À votre avis, quels sont les facteurs de risque associés à la dyslipidémie chez les enfants ? (Cochez toutes les réponses qui s'appliquent)

    a) Antécédents familiaux de maladies cardiaques
    b) Régime alimentaire riche en graisses
    c) Manque d'activité physique
    d) Obésité
    e) Consommation élevée de sucre
    f) Autre (veuillez préciser) :

4. À votre connaissance, quelles maladies peuvent être causées par la dyslipidémie ? (Cochez toutes les réponses qui s'appliquent)

    a) Infarctus du myocarde (crise cardiaque)
    b) Athérosclérose (dépôt de graisses dans les vaisseaux sanguins)
    c) Stéatose hépatique (foie gras)
    d) Diabète
    e) Pancréatite aiguë (inflammation du pancréas)

5. Selon vous, comment la dyslipidémie est-elle diagnostiquée ?

    a) Par une analyse sanguine
    b) Par un examen clinique
    c) Par un test génétique
    d) Par une évaluation du régime alimentaire
    e) Je ne sais pas

6. À votre avis, quelles sont les stratégies de prise en charge de la dyslipidémie chez les enfants que vous connaissez ? (Cochez toutes les réponses qui s'appliquent)

   a) Modifications du régime alimentaire
   b) Augmentation de l'activité physique
   c) Médication
   d) Surveillance régulière du profil lipidique
   e) Conseils sur le mode de vie
   f) Je ne sais pas
   g) Autre (veuillez préciser) :

7. Selon vous, quelles mesures préventives peuvent aider à réduire le risque de dyslipidémie chez les enfants ? (Cochez toutes les réponses qui s'appliquent)

   a) Je ne sais pas
   b) Habitudes alimentaires saines
   c) Exercice physique régulier
   d) Limitation du temps passé devant les écrans
   e) Bilans de santé réguliers
   f) Éducation sanitaire pour les enfants
   g) Autre (veuillez préciser) :

Partie 4. Pratiques de dépistage et habitudes de prévention

1. Avez-vous déjà fait dépister vos enfants pour la dyslipidémie par une analyse sanguine ?

   a) Oui
   b) Non

2. Si oui, pourriez-vous indiquer la date et les résultats de la dernière analyse de vos enfants ?

Date:

Résultats:

- Cholestérol : mg/dL ou mmol/l
- Triglycérides : mg/dL ou mmol/l
- HDL-Cholestérol: mg/dL ou mmol/l

3. Vos enfants reçoivent-ils un traitement pour la dyslipidémie ?

   a) Oui
   b) Non

4. Encouragez-vous vos enfants à être physiquement actifs ?

   a) Oui
   b) Non

5. Si oui, à quelle fréquence ?

   a) Quotidiennement
   b) Une fois par semaine
   c) Deux fois par semaine
   d) Trois fois ou plus

6. Fournissez-vous à vos enfants une alimentation équilibrée ?

   a) Jamais
   b) Rarement
   c) Parfois
   d) Toujours

7. Fournissez-vous à vos enfants des légumes et des fruits ?

   a) Tous les jours
   b) Deux fois par semaine
   c) Une fois par semaine
   d) Jamais

8. Seriez-vous intéressé(e) par davantage d'informations sur la dyslipidémie chez les enfants ?

   a) Oui
   b) Non

9. Quels types de ressources vous seraient les plus utiles ? (Cochez toutes les réponses qui s'appliquent)

   a) Brochures d'information
   b) Ateliers ou séminaires
   c) Ressources en ligne
   d) Consultations individuelles avec des professionnels de santé
   e) Programmes scolaires
