# Supplementary material for: Dyslipidemia in Lebanese children and adolescents: parents’ knowledge and practices
Source: BMC Public Health. 2026 Jun 24;26:2801. doi: 10.1186/s12889-026-28254-8 (PMC13637342; doi:10.1186/s12889-026-28254-8)
Supplement: Supplementary file 3 [file 12889_2026_28254_MOESM3_ESM.pdf]

I. Arabic version:

ورقة معلومات

السيد/السيدة المحترم/ة،

اسمي خليل حمادة وأنا طالب في السنة السابعة في كلية الطب في جامعة القديس يوسف، أعمل على أطروحة تحت إشراف البروفيسورة ماري-هيلين غناجي-يارد

(Email : mariehelene.yared@usj.edu.lb, tel. : 03291301) .

أود دعوتكم للمشاركة في مشروع بحثي بعنوان **"خلل شحميات الدم لدى الأولاد في لبنان: معرفة وممارسات الآباء"**، والذي يتعلق بدراسة معرفة وممارسات الآباء بشأن هذه المشكلة الصحية لدى الأولاد.

تمت الموافقة على هذا المشروع من قبل لجنة الأخلاقيات في جامعة القديس يوسف.

يُعرّف خلل شحميات الدم بأنه مستوى غير طبيعي من الدهون في الدم (الكوليسترول، الدهون الثلاثية، الكوليسترول عالي الكثافة). إنه شائع بين الأطفال والمراهقين وأصبح مشكلة صحية عامة في السنوات الأخيرة. عدد الأطفال والمراهقين الذين تم تشخيصهم بهذه الحالة في تزايد. في لبنان، تكون النسبة مرتفعة بشكل خاص، حيث تؤثر على طفل واحد من بين كل أربعة أطفال. يزيد خلل شحميات الدم لدى الأطفال والمراهقين من خطر الإصابة بأمراض القلب والأوعية الدموية في مرحلة البلوغ، لذلك يمكن أن يمنع الفحص المبكر لهذا الاضطراب أمراض القلب والأوعية الدموية في مرحلة البلوغ.

مشاركتكم في هذا الاستبيان **طوعية**. من خلال المشاركة في هذه الدراسة، ستقدمون لنا خدمة كبيرة وتمكنوننا من معرفة المزيد عن إدارة خلل شحميات الدم لدى الأطفال في لبنان ومواقف الآباء تجاه هذا الموضوع. ستكون مشاركتكم أساسية في تحسين فهمنا لهذا الموضوع وتعزيز أنماط الحياة الصحية.

يحترم هذا الاستبيان **خصوصيتكم** وسيتم التعامل مع محتوى إجاباتكم **بأقصى درجات السرية**.

أنتم أحرار في الإجابة على أي أو جميع الأسئلة. شكراً لكم مسبقاً على وقتكم.

لمزيد من المعلومات، يرجى الاتصال بخليل حمادة:

(Email : khalil.hamadeh@net.usj.edu.lb, tel. : 71210807) .

الاستبيان

الجزء الأول: الخصائص الديموغرافية والشخصية

1. **العمر**

2. **الجنس :**ذكر / أنثى

3. **المستوى التعليمي:**

a) مدرسة ابتدائية
b) مدرسة ثانوية
c) جامعة

4. **الوضع المهني:**

a) عامل في القطاع الصحي
b) غير ذلك

5. **كم عدد الأطفال لديك؟ ---**

الجزء الثاني: وعي الأهالي باضطراب دهون الدم لدى الأطفال

1. **هل سمعت من قبل عن اضطراب دهون الدم (اضطرابات الدهون) لدى الأطفال؟**

a) نعم
b) لا

2. **إذا كانت الإجابة بنعم، كيف سمعت عن هذه المشكلة لأول مرة؟**

a) من خلال أخصائي صحي (طبيب، ممرض...)
b) في المدرسة
c) من وسائل الإعلام (تلفزيون، إنترنت، إلخ)
d) من العائلة أو الأصدقاء
e) أخرى (يرجى التحديد):

3. **كيف تصف مستواك الحالي من المعرفة حول اضطراب دهون الدم لدى الأطفال؟**

a) معرفة جيدة جداً
b) معرفة متوسطة
c) معرفة قليلة
d) لا توجد معرفة

الجزء الثالث: معرفة الأهالي باضطراب دهون الدم لدى الأطفال

1. **هل تعتقد أنه ينبغي فحص أطفالك للكشف عن اضطراب دهون الدم؟**

a) نعم
b) لا

2. **في أي عمر تعتقد أن الفحص للكشف عن اضطراب دهون الدم يجب أن يبدأ؟**

a) من 9 إلى 11 سنة
b) 21 سنة فما فوق
c) 30 سنة فما فوق

d) فقط في حال وجود مشاكل طبية

3. **في رأيك، أي من عوامل الخطر التالية ترتبط باضطراب دهون الدم لدى الأطفال؟ (اختر كل ما ينطبق)**

a) تاريخ عائلي من أمراض القلب
b) نظام غذائي غني بالدهون
c) قلة النشاط البدني
d) السمنة
e) الإفراط في تناول السكريات
f) أخرى (يرجى التحديد):

4. **حسب معرفتك، ما هي الأمراض التي يمكن أن يسببها اضطراب دهون الدم؟ (اختر كل ما ينطبق)**

a) احتشاء عضلة القلب (النوبة القلبية)
b) تصلب الشرايين (تراكم الدهون في الأوعية الدموية)
c) تشحم الكبد (الكبد الدهني)
d) داء السكري
e) التهاب البنكرياس الحاد

5. **كيف تعتقد أنه يتم تشخيص اضطراب دهون الدم؟**

a) عن طريق فحص الدم
b) عن طريق الفحص السريري (الفيزيائي)
c) عن طريق الفحوصات الجينية
d) عن طريق تقييم النظام الغذائي
e) لا أعرف

6. **في رأيك، ما هي استراتيجيات التعامل مع اضطراب دهون الدم لدى الأطفال التي تعرفها؟ (اختر كل ما ينطبق)**

a) تغييرات في النظام الغذائي
b) زيادة النشاط البدني
c) العلاج بالأدوية
d) المراقبة الدورية لمستوى الدهون في الدم
e) نصائح وإرشادات حول نمط الحياة
f) لا أعرف
g) أخرى (يرجى التحديد):

7. **في رأيك، ما هي الإجراءات الوقائية التي يمكن أن تساعد في تقليل خطر إصابة الأطفال باضطراب دهون الدم؟ (اختر كل ما ينطبق)**

a) لا أعرف
b) عادات غذائية صحية
c) ممارسة التمارين الرياضية بانتظام
d) الحد من الوقت المستهلك أمام الشاشات
e) الفحوصات الطبية الدورية
f) التثقيف الصحي للأطفال

g) أخرى (يرجى التحديد):

الجزء الرابع: ممارسات الفحص وعادات الوقاية

1. **هل قمت يوماً بفحص أطفالك للكشف عن اضطراب دهون الدم باستخدام تحليل الدم؟**

a) نعم
b) لا

2. **إذا كانت الإجابة بنعم، هل يمكنك تحديد تاريخ ونتائج آخر تحليل لأطفالك؟**
   - **التاريخ:**
   - **النتائج:**
     - الكوليسترول (Cholesterol): .... ملغ/دسل أو ممول/ل
     - الدهون الثلاثية (Triglycerides): .... ملغ/دسل أو ممول/ل
     - الكوليسترول الجيد (HDL-Cholesterol): .... ملغ/دسل أو ممول/ل

3. **هل يتلقى أطفالك علاجاً لاضطراب دهون الدم؟**

a) نعم
b) لا

4. **هل تشجع أطفالك على ممارسة النشاط البدني؟**

a) نعم
b) لا

5. **إذا كانت الإجابة بنعم، كم مرة؟**

a) يومياً
b) مرة في الأسبوع
c) مرتين في الأسبوع
d) ثلاث مرات أو أكثر

6. **هل تقدم لأطفالك نظاماً غذائياً متوازناً؟**

a) أبداً
b) نادراً
c) أحياناً
d) دائماً

7. **هل تقدم لأطفالك الخضار والفواكه؟**

a) كل يوم
b) مرتين في الأسبوع

c) مرة في الأسبوع
d) أبداً

8. **هل تود الحصول على مزيد من المعلومات حول اضطراب دهون الدم لدى الأطفال؟**

a) نعم
b) لا

9. **ما هي نوعية المصادر والموارد التي تجدها أكثر فائدة لك؟ (اختر كل ما ينطبق)**

a) كتيبات ونشرات معلوماتية
b) ورش عمل أو ندوات
c) مصادر ومواقع عبر الإنترنت
d) استشارات فردية مع أخصائيين صحيين
e) برامج مدرسية
